# Experimentally Probing the Effect of Confinement Geometry on Lipid Diffusion

**DOI:** 10.1021/acs.jpcb.3c07388

**Published:** 2024-04-04

**Authors:** Nicole Voce, Paul Stevenson

**Affiliations:** Department of Physics, Northeastern University, Boston, Massachusetts 02115, United States

## Abstract

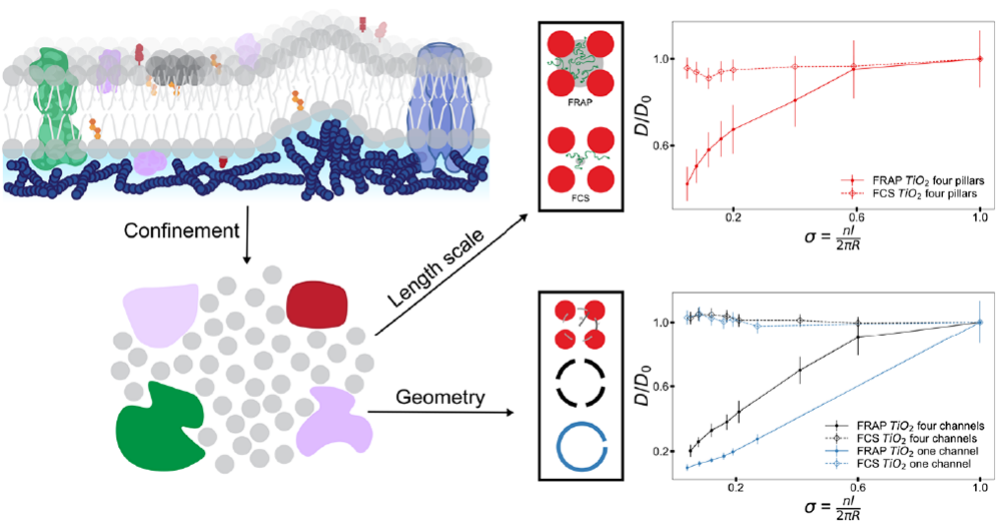

The lateral mobility of molecules within the cell membrane is ultimately governed by the local environment of the membrane. Confined regions induced by membrane structures, such as protein aggregates or the actin meshwork, occur over a wide range of length scales and can impede or steer the diffusion of membrane components. However, a detailed picture of the origins and nature of these confinement effects remains elusive. Here, we prepare model lipid systems on substrates patterned with confined domains of varying geometries constructed with different materials to explore the influences of physical boundary conditions and specific molecular interactions on diffusion. We demonstrate a platform that is capable of significantly altering and steering the long-range diffusion of lipids by using simple oxide deposition approaches, enabling us to systematically explore how confinement size and shape impact diffusion over multiple length scales. While we find that a “boundary condition” description of the system captures underlying trends in some cases, we are also able to directly compare our systems to analytical models, revealing the unexpected breakdown of several approximate solutions. Our results highlight the importance of considering the length scale dependence when discussing properties such as diffusion.

## Introduction

**Figure 1:**
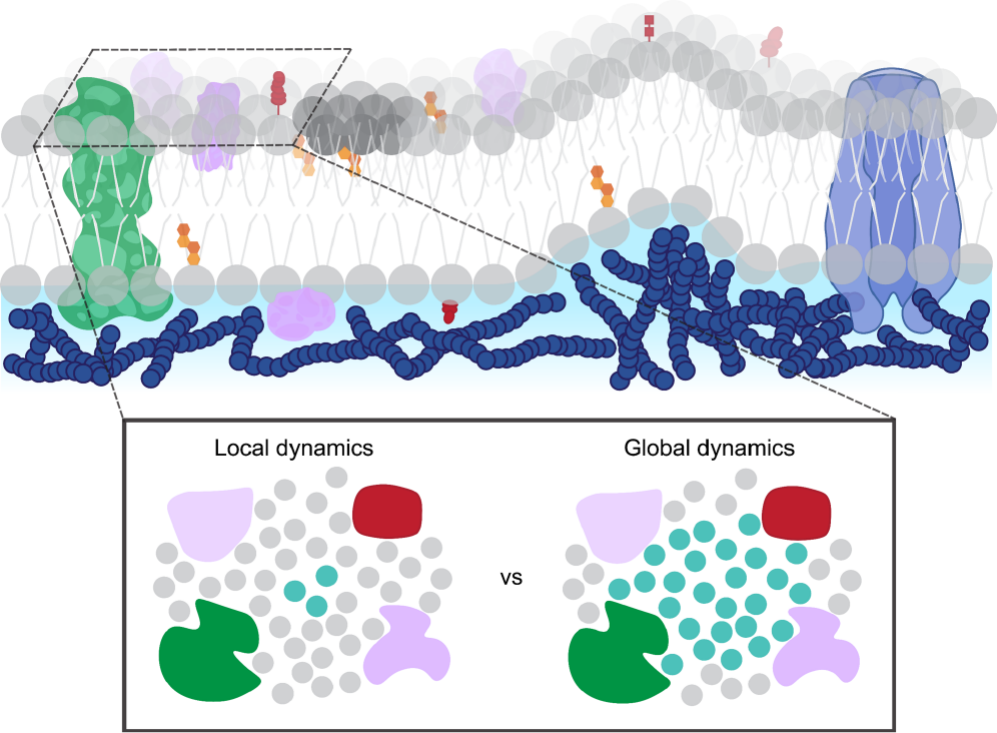
Membranes are crowded, congested environments with obstructions spanning length scales from nanometers to microns. Schematic showing a membrane domain with a transmembrane protein (green), integral and peripheral proteins (purple), cholesterol (orange/red), glycosphingolipids (red), actin meshwork (navy), an ion channel (blue), and a lipid aggregate (dark gray). In this work, we probe the effect of the confinement geometry on both local and global scales.

The cell membrane is a crowded, continuously fluctuating environment composed of a diverse population of lipids, proteins, and small molecules (Figure 1). Characterizing and understanding the dynamics of these components and how they interact are crucial to developing an effective description of both structural and functional aspects of the membrane. This has motivated extensive experimental and theoretical efforts to probe the motion of lipids over a wide range of length scales.^1−5^ However, the complex, heterogeneous nature of biological membranes makes systematic exploration of the energy landscape of systems *in vivo* extremely challenging. Of particular importance is understanding how the effect of confinement manifests in these systems and shapes the behavior of membrane components. Confinement can influence the diffusion of small molecules in a wide range of contexts^6−12^ spanning many length scales; one important example is the localization of membrane components by actin filaments, where domain sizes range from nanometer to micrometer scales.^13−17^ Hierarchical, scale-dependent behavior has been observed in these networks,^18^ having a range of geometries, spanning mesh-like irregular networks^19^ to ordered rings.^17^ This range of length scales and geometries motivates efforts to understand the driving forces between specific molecular interactions and physical boundary conditions, how this impacts processes such as diffusion and binding, and how this depends on the length scale.

Diffusion plays a key role in many biological processes, ultimately determining the rate of encounter of membrane proteins and mass transport within the membrane. Developing a picture of how diffusion changes in response to different membrane perturbations is therefore of great importance, which has motivated many efforts at characterizing diffusion of systems such as particles in the presence of obstacles,^6,11,20−26^ membrane flow under different conditions,^27,28^ and components in curved membranes.^29−31^ The ubiquity of confined environments in biological systems, such as ions in ion channels,^32^ molecules in dendritic spines,^33^ and ligands binding to cells,^34^ has also motivated extensive theoretical descriptions of diffusion in these systems,^35−45^ which have robustly demonstrated the nontrivial relationship between diffusion and geometry.^46−50^ In many cases, however, the validity of these descriptions has not been experimentally tested, leaving unanswered questions about when specific molecular interactions can be neglected, when a confined membrane should be described as a continuous fluid or discrete particles, or how to treat irregularly shaped confined regions. Even in the simplest systems – a membrane composed of lipids, with no proteins or other small molecules – challenges remain. Simulating diffusion in these systems is substantially more complicated than it first appears^51,52^ even in the absence of confinement because of subtle artifacts in finite-size simulations.

Here, we systematically explore how the effects of geometric confinement shape and control the motion of lipids in membranes at different length scales by using thin oxide structures to form easily fabricated obstacles. Using a combination of fluorescence recovery after photobleaching (FRAP) and fluorescence correlation spectroscopy (FCS) approaches, we characterize how these systems can be described globally in terms of “boundary conditions,” which depend on the geometry of confinement, while still locally retaining their native diffusion coefficient, revealing the complex scale-dependent behavior of these dynamics.

## Materials and Methods

### Vesicle Preparation

Vesicles used in this study were prepared from 1,2-dilauroyl-*sn*-glycero-3-phosphocholine (DLPC) and the label 1,2-dipalmitoyl-*sn*-glycero-3-phosphoethanolamine-*N*-(lissamine rhodamine B sulfonyl) (Rhod-PE), both purchased from Avanti Polar Lipids (Alabaster, AL). Prior to vesicle formation, Rhod-PE was stored in chloroform, DLPC in its powder form, and all lipids were stored at −20 °C. For FRAP measurements, a 1.2 mg/mL DLPC 0.5 mol % Rhod-PE solution was prepared by mixing appropriate amounts of DLPC, Rhod-PE, and chloroform (≥99%; Sigma-Aldrich; Darmstadt, Germany) and then dried under N_2_ gas and placed under vacuum for 24 h. The lipid film was rehydrated with phosphate-buffered saline (Boston BioProducts Inc.; Ashland, MA), and large unilamellar vesicles (LUVs) were formed by extruding through a 0.1 μm pore size polycarbonate membrane a total of 21 times. The mini-extruder set used to form LUVs was purchased from Avanti Polar Lipids. For FCS measurements, a 0.3 mg/mL DLPC 0.0025 mol % Rhod-PE solution was prepared following the same procedure.

### Substrate Preparation

SiO_2_ substrates were formed by dicing wet thermal oxide wafers (University Wafers; South Boston, MA) into 1 cm × 1 cm chips. Substrates were subsequently cleaned by sonicating in 1:1 IPA:acetone for 2 min and then dried under N_2_ gas. Residual organic contaminants were removed by exposing the substrate to oxygen plasma treatment for 2 min (Anatech SP-100 Plasma System). Supported lipid bilayers were formed on the substrates immediately after the oxygen plasma cleaning using the vesicle fusion method, described in the following section.

TiO_2_-patterned substrates were formed by using photolithography. First, wet thermal oxide wafers were exposed to oxygen plasma treatment for 2 min. Immediately after the oxygen plasma cleaning, they were coated in a thin layer of S1813 photoresist by spinning at 4000 rpm for 1 min and then baking at 115 °C for 1 min. A quartz mask coated in chrome was used to pattern the photoresist. A Quintel 4000 Mask Aligner was used to expose the wafer for 7 s. The pattern was developed in AZ 726 MIF for 45 s, then rinsed with DI water for 1 min, and dried under N_2_ gas. The developed wafer was plasma-cleaned for 1 min. Using the Savannah 100 system, ∼4 nm of TiO_2_ (atomic force microscopy characterization shown in Figure S1) was grown on top of the wafer through atomic layer deposition (ALD) at 105 °C. Low-temperature deposition provides a cleaner liftoff, avoiding the potential destruction or outgassing of the resist layer.^53^ After ALD, the S1813 photoresist was stripped by sonicating the wafer in acetone for 2 min and then drying under N_2_ gas, leaving a SiO_2_ substrate with TiO_2_ obstacles ∼4 nm in height. To investigate the material dependence, Al_2_O_3_-patterned substrates were made in the same way. Immediately before bilayer formation, the substrates were cleaned in 1:1 IPA:acetone (sonicating for 2 min) and then exposed to oxygen plasma treatment for 30 s.

**Figure 2:**
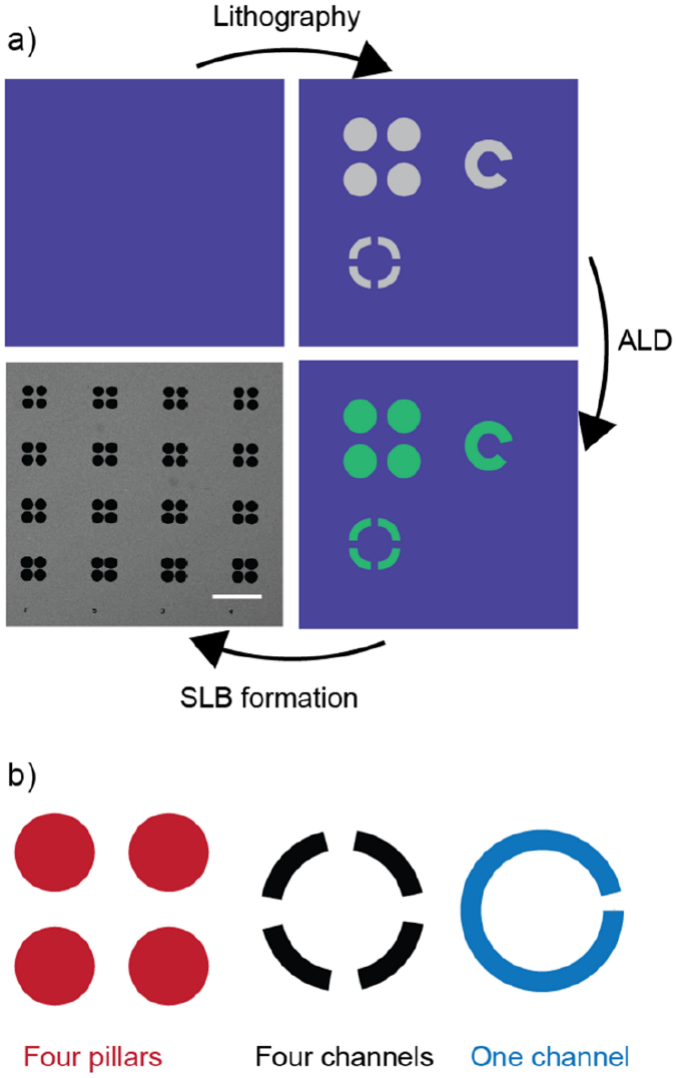
(a) Schematic of the fabrication process, starting with a plain SiO_2_ substrate. The SiO_2_ substrate is lithographically patterned, developed, and then 2.5–5 nm of TiO_2_ or Al_2_O_3_ is deposited on the surface through atomic layer deposition (ALD). The substrate is cleaned, and supported lipid bilayers (SLBs) are formed on the SiO_2_ surfaces with the vesicle fusion method. The bottom left panel is a fluorescence image showing a SLB (dark gray) formed on a SiO_2_ substrate around TiO_2_ structures (black circles). Scale bar is 100 μm. (b) Three classes of structures used to explore geometric aspects of confinement.

The patterned substrates host three families of geometries: four circular pillars in a square array (termed “four pillars”), four arcs arranged in a circular array (“four channels”), and a continuous arc forming an almost-complete circle (“one channel”). These are shown schematically in Figure 2b. SiO_2_, TiO_2_-patterned, and Al_2_O_3_-patterned substrates were adhered to a CoverWell imaging gasket (Thermo Fisher Scientific; Waltham, MA) with double-sided Scotch tape.

### Supported Lipid Bilayers

Supported lipid bilayers (SLBs) were formed using the vesicle fusion method, described elsewhere.^54^ Briefly, the TiO_2_-patterned substrates were placed on a hot plate held at 30 °C, well above the gel–fluid phase transition temperature of DLPC (−1 °C).^55^ 30 μL of the LUVs was deposited on the substrates, followed by 90 μL of phosphate-buffered saline. The LUVs were left to incubate on the substrates for 10 min. After incubation, 700 μL of phosphate-buffered saline was added to the gasket. The substrate was washed with phosphate-buffered saline a total of 15 times. At the end of the washing step, a 30 × 22 mm coverslip (Thermo Fisher Scientific; Waltham, MA) was rolled on top of the substrate. For FCS measurements, the sides of the gasket were coated with clear nail polish (Sally Hanson Xtreme-Wear; CVS) on top to avoid sample drying. A step-by-step protocol for forming SLBs with the vesicle fusion method can be found in archived protocols online.^56^ The full fabrication process is illustrated in Figure 2a.

### Fluorescence Recovery After Photobleaching (FRAP)

**Figure 3:**
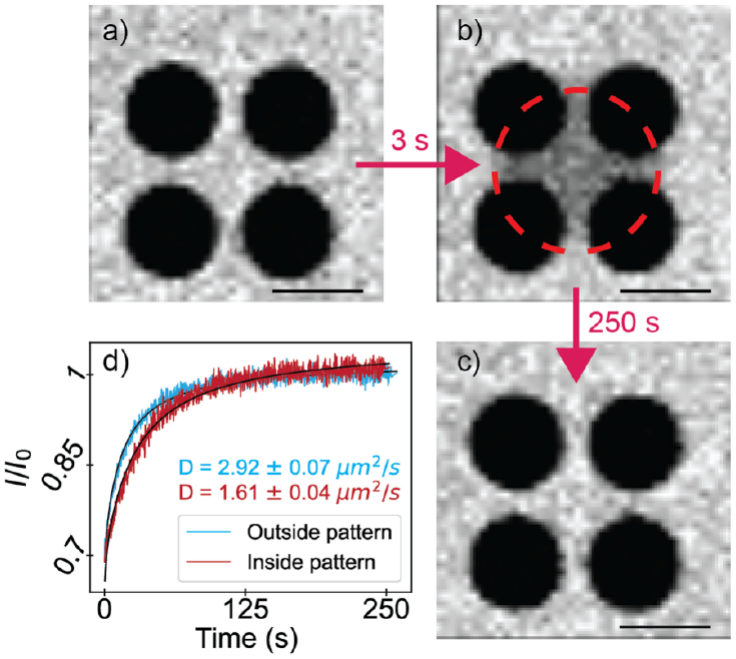
FRAP enables imaging of the bilayer morphology and fluidity. Bilayers readily form and recover on SiO_2_ substrates, as shown in (a)–(c). (d) Recovery of fluorescence inside (red) and outside (blue) the pattern. Outside the pattern, the diffusion coefficient is consistent with literature values for other one-phase fluid bilayers at room temperature^4,150,151^ (2.92 ± 0.07 μm^2^/s). Inside the pattern, the diffusion coefficient decreases; in the geometry shown, it decreases to 1.61 ± 0.04 μm^2^/s. The scale bar is 15 μm.

FRAP measurements are discussed elsewhere in detail.^57^ Here, SLBs were immediately imaged after formation with a Zeiss LSM 880 fluorescence microscope equipped with a 10× objective and a 561 nm diode laser (Oberkochen, Germany). Selected regions inside of the TiO_2_ geometries were bleached and monitored with the 561 nm laser (Figure 3; bleached radius 12.25 μm). The intensity of the bleached region was integrated and fit to obtain a characteristic diffusion time using eq 1:^58^
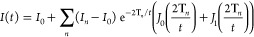
1

where *I*_0_ is the fluorescence intensity just after the bleach, *J*_0_ and *J*_1_ are the modified Bessel functions of orders 0 and 1, respectively, *I*_*n*_ is the intensity contribution of species *n* at *t* = ∞, and *T*_*n*_ is the characteristic diffusion time of species *n*. In our experiments, satisfactory fits are given for *n* = 1. Eq 1 is derived for a system without obstructions; our use of this analysis here is motivated by the successful application of this equation in previous studies exploring obstructed diffusion,^21,59−61^ and we note the agreement between this functional form and our experimental data (Figure 3d and Supporting Information). Diffusion coefficients were calculated using the following equation:
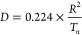
2

where *R* is the radius of the bleached region.

Data were collected for all geometries on one patterned substrate at a time. Each substrate had at least three repetitions of the patterns shown in Figure S2. One FRAP data collection consisted of measuring the diffusion within all geometries for each pattern; this was repeated twice for two different patterns on each substrate. Data were recorded in one batch for each substrate. Data were collected on three different substrates on three different days. All diffusion data were normalized with respect to diffusion measured outside of the patterns to account for the systematic differences in the environment.

### Fluorescence Correlation Spectroscopy (FCS)

FCS measurements were carried out using a 532 nm diode-pumped solid-state laser (GEM, Novanta Photonics; Bedford, MA) at a power less than 100 μW in a confocal microscope setup described in the Supporting Information. Briefly, the 532 nm laser beam was used to illuminate a 100× (1.25 NA) oil immersion objective (Zeiss; Oberkochen, Germany). Fluorescence was collected through the objective, and the emission was filtered by a 550 nm long-pass dichroic mirror (Thorlabs; Newton, NJ). A single-mode fiber acted as a pinhole. The fluorescence signal was detected by an avalanche photodiode (Excelitas Technologies; Waltham, MA) coupled to the fiber and was correlated with the Time Tagger Series software from Swabian Instruments (Time Tagger Ultra, Swabian Instruments; Stuttgart, Germany). The FCS setup was calibrated using 10 nM solutions of Rhodamine 6G (TCI America; Portland, OR) (Rhod-6G) in Millipore water following previously established calibration procedures.^62,63^

Measurements were taken by positioning the laser focal spot in the center of the TiO_2_ geometries. Each acquisition consisted of 10 FCS measurements of 5 s duration recorded at that position. The correlation functions were fit with a 2D diffusion model:
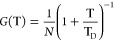
3where *N* is the average molecule number in the detection volume, *Τ* is the lag time, and *T*_D_ is the average time of molecules diffusing through the detection volume.^62,64^ FCS measurements were taken in the middle of each TiO_2_ geometry twice at different spatial locations on the TiO_2_-patterned substrates. The resultant *T*_D_ and *N* were averaged for each pattern, and the diffusion coefficient was calculated from the subsequent average *T*_D_ using the following equation:
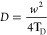
4where *w =* 191 ± 7.42 nm is the beam waist obtained from the Rhod-6G calibration measurements. The error in *w* was calculated from the standard deviation of the results of 10 calibration acquisitions.

### Numerical Simulation of Obstructed Diffusion

We simulate the expected FRAP response for different geometries by numerically solving the diffusion equation using VCell.^65,66^ To model the TiO_2_ obstacles, we applied zero-flux boundary conditions around regions matching the geometries in our experiment. We initially generate a circular profile of nonzero concentration (representing the bleached lipids) and allow this to propagate. We simulate our experiment by integrating the concentration in this region at each time step and fitting the resulting curve to extract the effective diffusion coefficient in the same way as the experimental data.

## Results and Discussion

### Effect of Confinement Geometry

**Figure 4:**
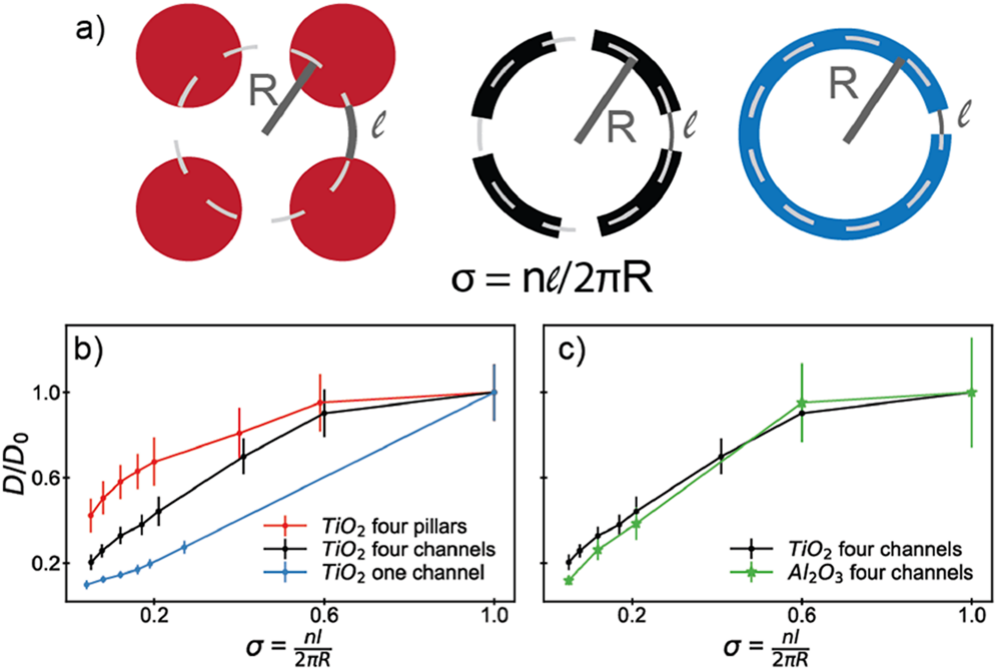
(a) The unobstructed fraction is defined as  where *n* is the number of escape channels, *l* is their arclength, and *R* is the radius of the bleached region (dashed gray circle). (b) The unobstructed fraction vs the effective diffusion for the three different TiO_2_ geometries are compared. (c) Comparison of the unobstructed fraction vs the effective diffusion for the four channel TiO_2_ structures on SiO_2_ (black circles) and for the four channel Al_2_O_3_ structures on SiO_2_ (green stars).

Figure 4b shows the effective diffusion of DLPC lipids as a function of unobstructed fraction for the three different geometries obtained by FRAP, which probes >μm length scales. The unobstructed fraction for each geometry is defined as
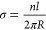
6

where *n* is the number of escape channels, *l* is the arc length of each escape channel, and *R* is the radius of the region shown schematically in Figure 4a. The FRAP data (Figure 4b) show a decrease in the effective diffusion coefficient as the unobstructed fraction decreases but also reveal a striking dependence on the geometry of the confining structure. Simply by changing the shape of the obstruction, we find that the micron-scale diffusion coefficient varies by a factor of up to four. Specifically, our FRAP data allow us to make two distinct observations: first, the pattern with a convex surface (“four pillars,” Figure 4b) shows a faster effective diffusion rate than either of the concave structures (“four channels” and “one channel,” Figure 4b); second, the arrangement of the escape channels (together in one channel or arranged regularly around the perimeter) impacts the effective diffusion, with a single larger channel giving rise to a *slower* effective diffusion than four smaller channels. The effect of confinement on the dynamics of membrane components has been well-studied in both experimental^15,16,19,20,22,25,57,59,67−77^ and theoretical contexts.^10,78,79^ Intuitively, it has been found that as the environment becomes increasingly crowded, the effective diffusion rate decreases,^7,11,25,26,80^ leading to a common model for obstructed diffusion in FRAP experiments framed in terms of the average obstructed fraction.^81−83^ Our observations are consistent with this general trend but also highlight the importance of not only the obstructed fraction but also the geometry of the obstacles when trying to extract the detailed dynamics of these systems.

In our experiments, we irradiated a constant area to bleach the fluorescent lipid and monitored the recovery. In the case of the four pillar geometry, however, this means that the lipid area in our probed region decreases as the radii of the pillars increase. We can consider the case where we scale the geometry and bleach radius to maintain a constant irradiated lipid area; here we expect the recovery time to scale as *R*^2^ (eq 1) for the unobstructed case, and our numerical simulations demonstrate that this also holds for our confined geometries (Supporting Information). Thus, we expect an appropriately normalized data set with varying pattern sizes to reproduce our data. However, this does lead to a potential explanation for the faster recovery of the four pillar geometry compared to the channel geometries for a given sigma; for a randomly diffusing lipid, the smaller free area of the four pillar geometry reduces the search area to find the exit.

The effect of concavity on confined dynamics has also previously been studied *in silico* in the context of membrane proteins and nanopores,^10,78^ though the effect of specific lipid-surface interactions in these cases makes it challenging to isolate the purely geometric effects. More recently, both experimental and theoretical descriptions of colloidal particles around curved interfaces have found concavity-dependent diffusion behaviors with slower diffusion around concave structures, consistent with our FRAP observations. We emphasize that experiments performed by Modica et al.^84^ probe fluorescent spheres several microns in diameter, many orders of magnitude different from our diffusing species, DLPC molecules, highlighting the broad importance of understanding the impact of geometry on confinement effects. However, even for a fixed degree of concavity, we also find that the effective diffusion coefficient depends on the arrangement of obstacles, as seen by comparing the clear variation in trends between the “four channel” and “one channel” data in Figure 4b.

### Global and Local Probes of Diffusion

**Figure 5:**
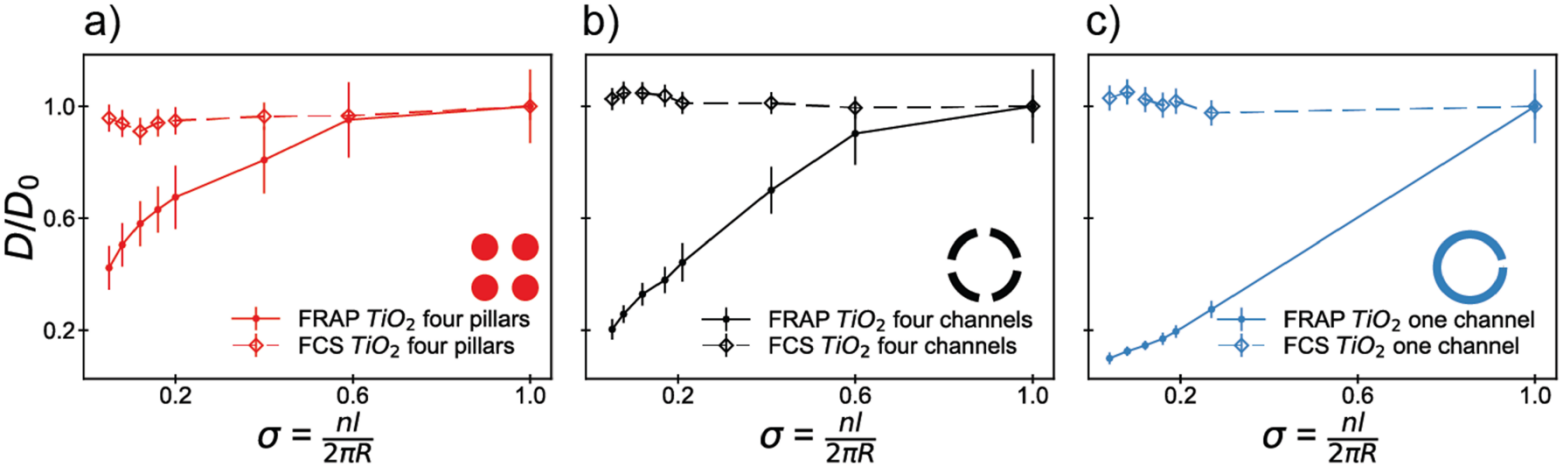
Effective diffusion for the three different TiO_2_ geometries (insets) obtained from FCS (diamonds) and FRAP (circles) measurements for (a) the four pillar geometry, (b) the four channel geometry, and (c) the one channel geometry.

To probe the effect of length scale on the observed diffusion behavior in our confined systems, we also performed FCS measurements at the center of each geometry. Figure 5 shows the effective diffusion of DLPC lipids as a function of the unobstructed fraction obtained from both FRAP and FCS measurements. FRAP probes the global diffusion of lipids on the many micron-scale whereas FCS probes more localized dynamics on the hundred-nanometer scale (here, <300 nm). The FCS data were collected at the center point of the confined domains; the FRAP data were collected for the entire confined domain.

We observed distinctly different behaviors with each of these probes. The local behavior in the center of the confined domains, as determined by FCS, does not depend on the pattern shape or the size of the channels in the pattern and is constant within our experimental precision; in all cases, the diffusion coefficient is statistically indistinguishable from a reference measurement taken far from any confining pattern (Figure 5). The FRAP data, however, show a clear decrease in the effective diffusion coefficient as the unobstructed fraction decreases. The equivalence of FCS and FRAP measurements for extracting diffusion coefficients has been demonstrated extensively,^4,85−90^ leading us to interpret our data in terms of length-scale dependent dynamics rather than any systematic difference between these two measurements. We assume that the diffusion measured by both FRAP and FCS is Brownian; we fit our FRAP data with the anomalous diffusion equation determined by Pastor et al.^91^ and found fit parameters converged toward α *=* 1 (free diffusion case; Figure S4). Additionally, the radius of the confined regions (*R* ∼ 12.25 μm; Figures 3a–c and 4a) is significantly larger than the FCS beam waist (*w* ∼ 191 nm), allowing our FCS measurements to be described by the free diffusion case.^92^ Differences between FRAP and submicron probes such as FCS or single-particle tracking can be observed in systems with heterogeneous environments, such as caveolae formation^93^ or picket-fence protein structures,^94−96^ where microscopic obstacles introduce length-scale dependent effective diffusion coefficients. We interpret the difference in our FCS and FRAP data in terms of free, unobstructed local diffusion (probed by FCS) and longer-range mass transport between the interior of the pattern and the outer lipid reservoir limited by the transit through the escape channels (probed by FRAP). Although FCS has higher spatial resolution than FRAP, in this case, we observe that FCS measurements are insensitive to the presence of obstacles. The FRAP data is collected over a larger area that includes the space near the domain boundaries, whereas the FCS data is collected in a small area in the *center* of the confined regions, rendering it agnostic to the existence of the domain walls. We interpret the observed differences in the FCS and FRAP data in terms of the inclusion of the portion of the bilayer near the domain boundaries in the FRAP measurements and the exclusion of this region in the FCS measurements. The opposite effect is observed by Calizo and Scarlata,^93^ where FCS measurements were sensitive to the presence of caveolae domains, but FRAP measurements were not. Here, the size of the obstacles is on the same length scale as our bleached region in the FRAP experiments (many microns) whereas caveolae domains are on the same length scale as the FCS beam diameter (hundred(s) of nanometers).^93^ However, similar effects to our data have been observed by Wawrezinieck et al. in simulating fully confined lipids, where diffusion slows as the probed length scale approaches that of the confining structure.^92^ This comparison highlights the importance of considering the length scale of the confinement or obstruction and choosing an approach that is sensitive to effects on that scale. Probing smaller length scales does not inherently provide greater sensitivity to all types of confinement, which will be important when considering micron-scale confinement possible with actin filaments.^17−19,23^

### Observed Trends Are Not Specific to TiO_2_

To explore whether the observed behaviors of the lipids are dependent on specific interactions with the TiO_2_ obstacles, we also generated systems using Al_2_O_3_ instead of TiO_2_. Both surfaces inhibit bilayer formation under our experimental conditions^97,98^ but differ in their surface chemistries and have different detailed interactions with supported lipid bilayers.^99−104^ Patterns from both materials show the same trends (Figures 4c and S6), indicating that the behaviors we see arise from nonspecific confinement geometry effects. The lack of material dependence motivates our description of confinement in terms of a general repulsive “boundary condition” imposed by the patterned material, as discussed below.

### Confinement as a Narrow-Escape Problem

The so-called “narrow escape” problem – where species confined in a region can exit only through a few discrete channels – has been studied theoretically in a vast range of different geometries,^35,36,40−43,45,105−109^ yet few of these have been directly tested against experiment. The confinement geometries used in our experiments were chosen, in part, to enable comparison with a range of theoretical and numerical approaches for calculating the effective diffusion of the lipids out of this region. Here, we directly compare our experimental data to analytical and numerical models commonly employed to describe the narrow escape problem to assess the correspondence with experiment.

**Figure 6:**
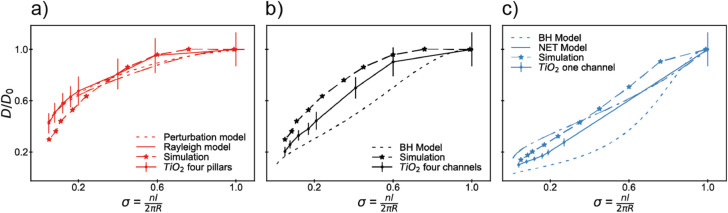
Comparison of experimental data with analytical models and numerical simulations for the various geometries in this work: (a) the four pillar geometry, (b) the four channel geometry, and (c) the one channel geometry. Descriptions of the models for each geometry are given in the main text.

In general, the narrow escape problem is formulated in terms of a local diffusion coefficient that is uniform across the sample region but where boundary conditions imposed by the confining structure impose an effective “global” diffusion coefficient limited by the exchange of species with the outside reservoir, consistent with our FRAP and FCS observations. Figure 6 shows the comparison between our experimental data and commonly used models from the literature, which describe our geometries. These models all describe the system in terms of a reflecting boundary (here, our TiO_2_ region) and an absorbing boundary (our escape channel) but differ in their approach to determining analytical expressions for these systems. Since we compare the diffusion normalized to the unconfined case, these models have no adjustable or system-specific parameters.

Interestingly, there is significant variation in the agreement between our experimental data and the results of the various models. For the four pillar geometry, we find that our numerical simulations, the perturbation theory-based approach of Mangeat et al.^105^ (perturbation model), and the multipole expansion-based approach of Rayleigh^107^ (Rayleigh model) agree with our FRAP data over the entire range of our experimental parameters (Figure 6a), with relatively minor variations between the expected effective diffusion coefficient. Numerical simulations of the diffusion also show the same general trend as our experimental data.

In contrast, the boundary-homogenization descriptions of Berezhkovskii and Barzykin^36^ (BH model) and the narrow escape problem for a circular disk^108−110^ (NET model) show marked deviations from our data both in quantitative value and in overall shape (Figure 6b,c). For the NET model, this is consistent with previous experimental observations, which show deviation from the model at σ > 0.05.^110^ The boundary homogenization approach, however, is expected to hold for all values of σ,^36^ but here we find that our experiments show different behavior for the entire experimental range. In contrast, our numerical simulations show qualitative agreement with the overall trend but consistently overestimate the effective diffusion of the confined lipid for the four channel and one channel structures.

One observation our comparison allows us to make is that the best agreement is observed in systems where zero-flux (Neumann) boundary conditions are employed (perturbation, Rayleigh models, and the numerical simulations), while the approach utilizing mixed boundary conditions (boundary homogenization) shows poorer agreement with our experimental results. However, the sensitivity of the boundary homogenization approach to the parametrization and functional form of the effective “trapping rate” provides an alternative explanation for the disagreement. Nevertheless, our results highlight not only the significant effect of confinement geometry on the diffusion of species in lipid membranes but also the need to experimentally test the limitations of analytical models developed, even for geometrically simple systems.

## Conclusion

We developed a novel lithography-based platform to study the diffusion of DLPC lipids inside confined regions. Global diffusion of lipids, probed by FRAP, was impeded inside the confined domains; the degree of this effect depends on the geometry of the domain but not on the material with which the domains were formed. This observation implies that the obstructed fraction is not the only parameter that determines how molecules diffuse in a crowded environment—the shape and form of the obstacles play a significant role in the diffusive behavior as well. We also demonstrate that the local effective diffusion of lipids in the center of the confined domain is the same as the effective diffusion of lipids in an obstacle-free membrane, emphasizing the importance of explicitly considering the length scale when describing dynamics. Lastly, the effective diffusion obtained from FRAP was compared with a range of theoretical models describing the system in terms of a repulsive “boundary condition.” Disagreement between the model for the concave geometries and the FRAP data indicates that there is underlying behavior not fully explained by the theoretical models.

## Data Availability

The data associated with this work are available at 10.5281/zenodo.10830128.

## Supporting Information Available

Additional information regarding the substrate preparation (Figures S1 and S2), FCS setup (Figure S3), appropriateness of FRAP fit (Figures S4 and S5), comparison between FRAP measurements for all TiO_2_ and Al_2_O_3_ geometries (Figure S6), and numerical simulations (Figures S7 and S8) (PDF)
