# Supplementary material for: Experimentally Probing the Effect of Confinement Geometry on Lipid Diffusion
Source: J Phys Chem B. 2024 Apr 4;128(18):4404–13. doi: 10.1021/acs.jpcb.3c07388 (PMC11089508; doi:10.1021/acs.jpcb.3c07388)
Supplement: Supplementary file 1 — jp3c07388_si_001.pdf [file jp3c07388_si_001.pdf]

Supplementary Information: Experimentally Probing the Effect of Confinement Geometry on Lipid Diffusion

Nicole Voce and Paul Stevenson
Department of Physics, Northeastern University, Boston, MA

SI Contents:

S1 – AFM Characterization of Patterned Structures

S2 – Fluorescent Micrographs of Patterned Bilayers

S3 – FCS Experimental Details

S4 – Brownian vs non-Brownian Diffusion Tests

S5 – FRAP Fitting Model

S6 – Material Dependence of Trends

S7 – Numerical Simulations

S1 – AFM Characterization of Patterned Structures:

We validated our fabrication process with AFM (Bruker ICON) measurements to characterize the topography of the structures. We determine a thickness of 4nm of the atomic-layer deposited TiO_2, with steep sidewalls and tall edge artifacts consistent with a single layer liftoff process, shown in Figure S1.

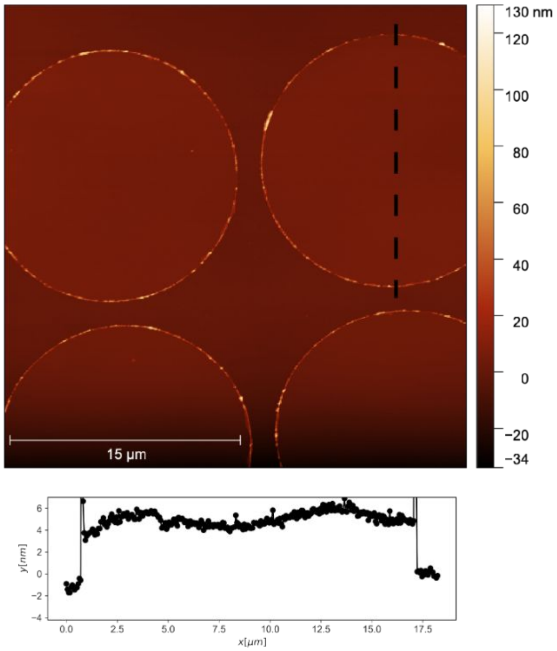

Figure S 1 *AFM image of a representative TiO_2 four pillar geometry. The ALD TiO_2 is roughly 4 nm in height with taller edges.*

S2 – Fluorescent Micrographs of Patterned Bilayers:

Figure S2 shows large-area fluorescent micrographs of the arrays of patterned bilayers used in the main text. The bilayers are highly uniform on the millimeter scale, except where the TiO_2 is present where we see a clear absence of the fluorescently labeled lipid.

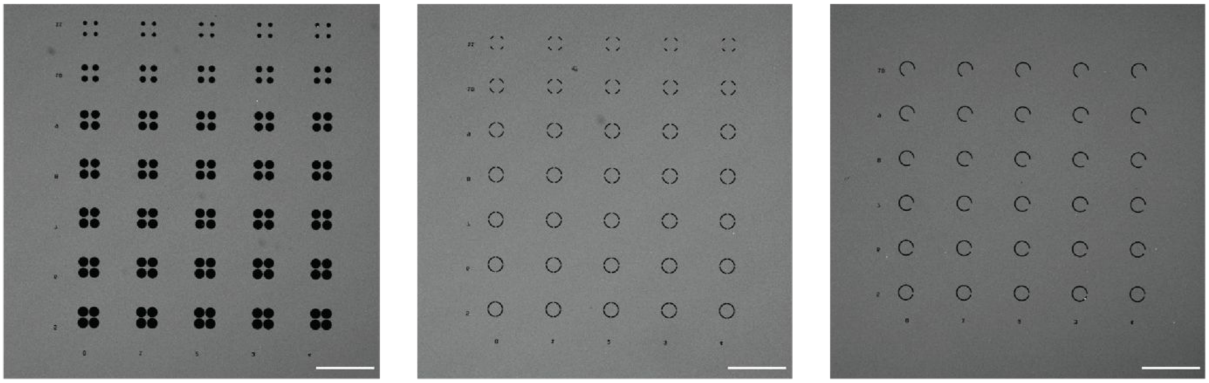

***Figure S 2** Fluorescence images showing a SLB (dark grey) formed on a SiO_2 substrate around all three TiO_2 geometries (black circles/arcs). Scale bars are 100 μm.*

S3 - FCS Experimental Details:

The FCS setup is illustrated in Figure S3. Before FCS data collection, the sample is imaged using a white LED light source and a camera (Basler AG; Ahrensburg, Germany) while the beam from the 532 nm diode-pumped solid-state laser (GEM, Novanta Photonics; Bedford, MA) is blocked. Following the yellow imaging light path in Figure S3, the light emitted from the LED is directed

with a 50-50 beam splitter to another, removable 50-50 beam splitter (Thorlabs; Newton, NJ) and then passed through the 100x (1.25 NA) oil immersion objective (Zeiss; Oberkochen, Germany). The light reflected off the sample then travels back through the 100x objective and the two beam splitters. The second, non-removable beam splitter directs the reflected light to a mirror which then directs it into the camera. The configuration of the camera allows us to find the TiO_2 patterns and ensure that we are collecting data in the middle of the confined geometries. After imaging, the white LED is turned off and the beam from the 532 nm laser is set to a power less than 100 μW. Following the green excitation path (Figure S3), the beam is expanded using a telescope created with a 25 mm lens and a 100 mm lens (Thorlabs; Newton, NJ). Then, it is directed with a series of mirrors until it reaches the 550 nm long-pass dichroic mirror (Thorlabs; Newton, NJ). During data collection, the removable beam splitter is removed; this component is only used when imaging the sample with the camera. The beam passes from the dichroic into the 100x objective, exciting the fluorescently tagged lipids. The tagged lipids emit ~580 nm light (shown in Figure S3 in red) that then passes through the 100x objective and the dichroic. The emitted light is then directed with a series of mirrors and passed through a 550 nm long-pass emission filter (Thorlabs; Newton, NJ) into a 10x objective which focuses it into a single-mode fiber that acts as a pinhole. The fiber is

coupled to an avalanche photodiode (APD) (Excelitas Technologies; Waltham, MA) which detects the emitted fluorescence signal. Then, the signal is correlated with the Time Tagger Series software from Swabian Instruments (Time Tagger Ultra, Swabian Instruments; Stuttgart, Germany).

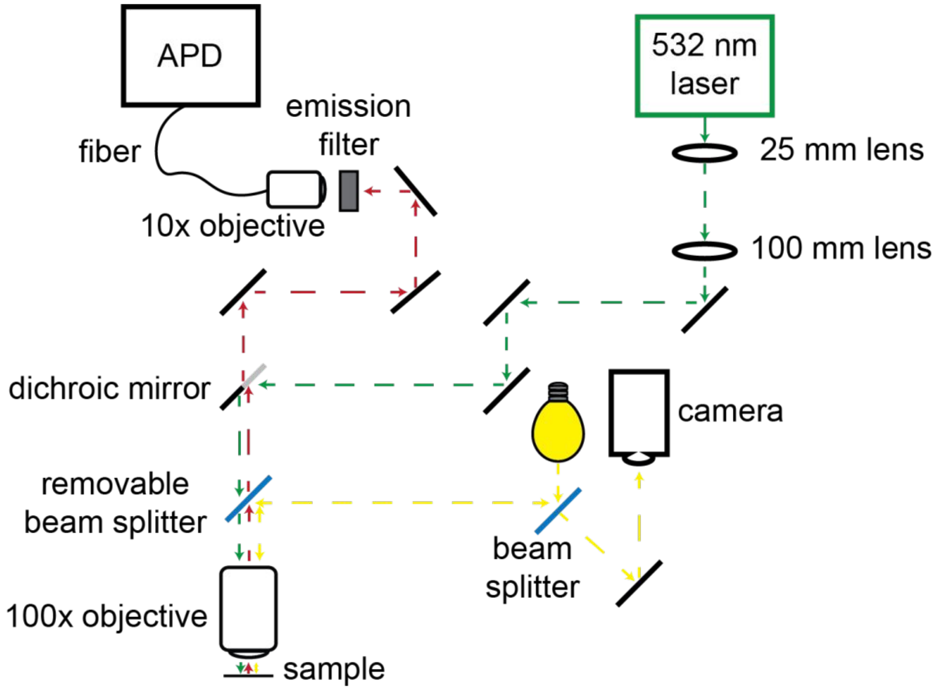

***Figure S 3** Schematic of FCS setup. The excitation light path is shown in green, the emission light path in red, and the imaging light path in yellow. The beam splitters illustrated are 50-50 beam splitters, the dichroic is a 550 nm long-pass filter, and the emission filter is a 550 nm long-pass filter. All unlabeled black lines represent mirrors.*

S4 – Brownian vs non-Brownian Diffusion Tests:

To determine whether our data requires a description which includes non-Brownian motion, we test for anomalous diffusion by fitting our data to the equation determined by Pastor et. al[1]:

$$I(t) = I_0 + \sum_n (I_n - I_0) e^{-2\left(\frac{T_n}{t}\right)^\alpha} \left(J_0\left(2\left(\frac{T_n}{t}\right)^\alpha\right) + J_1\left(2\left(\frac{T_n}{t}\right)^\alpha\right)\right)$$

Where I_0 is the fluorescence intensity just after the bleach, J_0 and J_1 are the modified Bessel functions of order 0 and 1, I_n is the intensity contribution of species n at $t = \infty$, T_n is the characteristic diffusion time of species *n,* and α is the anomalous coefficient. In our experiments, a single species diffuses, so we expect $n = 1$. We find that our data is best fit when $\alpha = 1$ (Figure S4), indicating that we are observing free diffusion ($0 < \alpha < 1$ is the case of subdiffusion and $\alpha > 1$ indicates superdiffusion). Based on these fits, we do not consider non-Brownian effects further in our analysis.

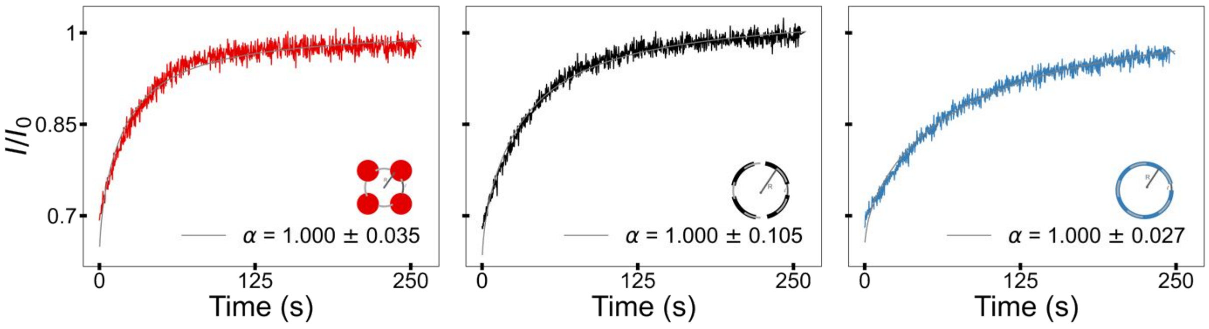

Figure S 4 *FRAP traces fit with the anomalous diffusion equation*[1]*, letting alpha be a free fitting parameter. FRAP traces shown were taken at* $\sigma \sim 0.2$ *for all* TiO_2 *geometries on* SiO_2

S5 –FRAP Fitting Model

The model used in the main text (Eq. 1) to fit the FRAP data is derived in the absence of obstructions; while we find good agreement between our experimental data and the model, we also consider possible artifacts. To ensure that the trends seen in Figure 5 were not artifacts of our FRAP fits, we analyzed our data using model-free metric—the time taken for the bleached fraction to recover half of its initial value ($T_{1/2}$). We find that the effective diffusion calculated from the FRAP fits agrees with the effective diffusion calculated from $T_{1/2}$ (Figure S5); both methods of analyzing the data give rise to the same trends.

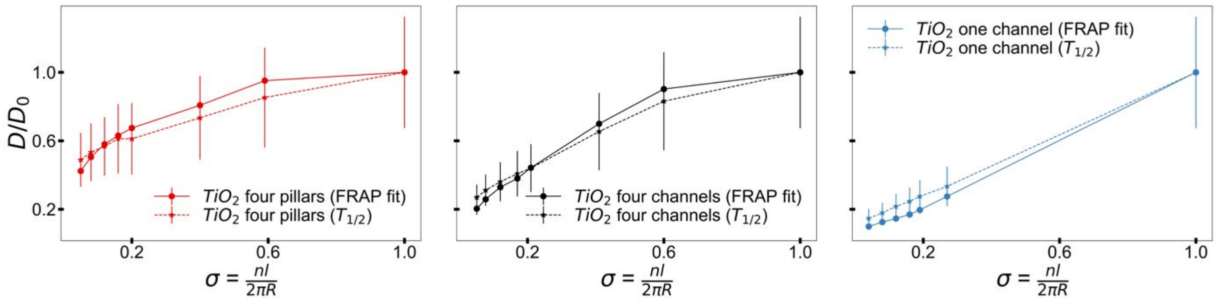

Figure S 5 *Compares the unobstructed fraction vs. the effective diffusion as calculated by fitting the FRAP intensity curves to Eq. 1 in the main text (circles) and as calculated by* $T_{1/2}$ *(stars) for all* TiO_2 *geometries on* SiO_2.

S6 – Material Dependence of Trends

We explore whether the choice of material used to create the diffusion obstacles causes the observed trends by repeating the same experiments using Al_2O_3 instead. Al_2O_3 also inhibits bilayer formation at neutral pH with the vesicle fusion method, but has a different surface charge, chemistry, structure, *etc.* We find that the observed trends in our data are consistent between these two materials.

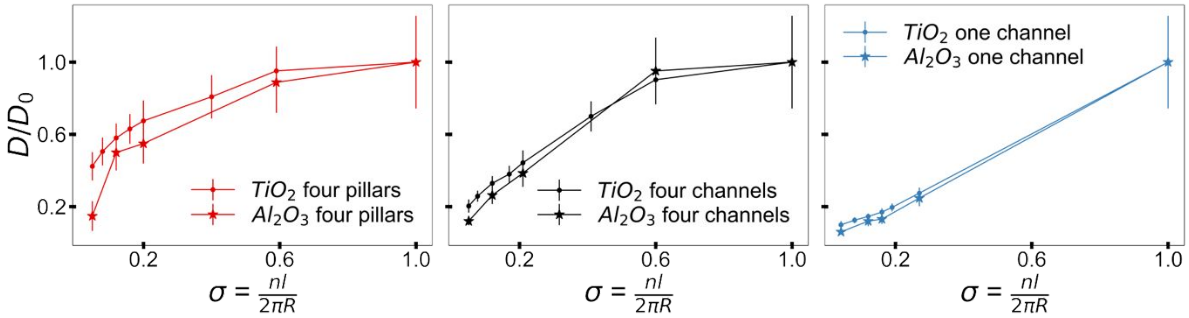

***Figure S 6** Comparison of the unobstructed fraction vs. the effective diffusion as measured by FRAP for all TiO_2 geometries on SiO_2 (circles) and all Al_2O_3 geometries on SiO_2 (stars).*

S7 - Numerical Simulations

Spatial diffusion

We use the implicit solver in VCell to solve the spatial and temporal dependence of the diffusion equation PDE for each geometry. Figure S7 shows the spatial profile at different times for one

particular four pillar geometry ($\sigma = 0.24$). To simulate FRAP traces, we integrate the region initially bleached in the simulation ($t = 0$ distribution) for each time point.

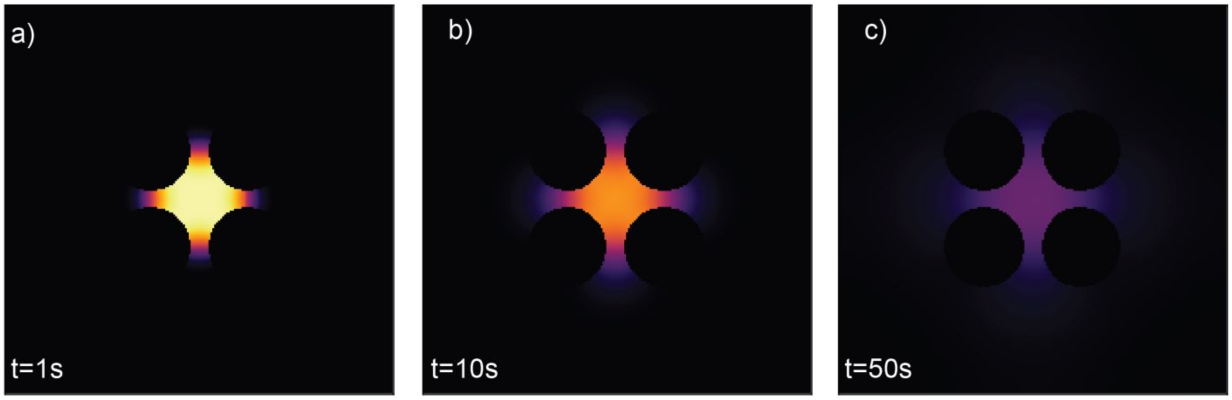

Figure S 7 *Simulated spatial profile of the bleached lipids for the four pillar, $\sigma = 0.24$ case.*

<u>Length scale dependence:</u>

To explore the effect of bleached volume on the recovery of the FRAP trace, we simulate our four pillar geometry at larger and smaller length scales compared to the experiment. For the unobstructed case, we expect the recovery time to increase as the square of the bleached radius; we find that this trend also holds in the confined geometries. Figure S8 shows traces for the

$\sigma = 0.085$ and $\sigma = 0.45$ cases, where, by scaling the time axes appropriately, we see the curves fall perfectly on top of each other.

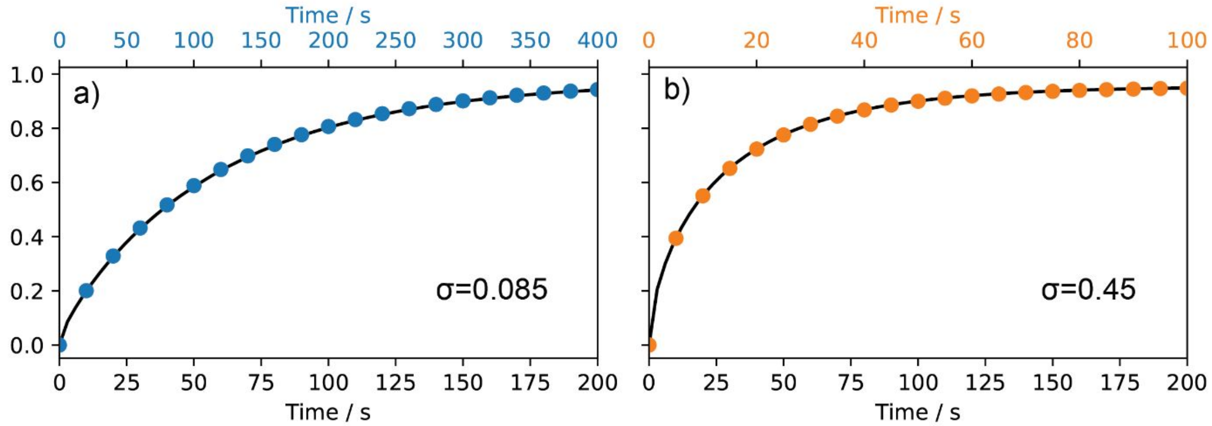

Figure S 8 *Simulated FRAP traces for two values of σ. The simulation is performed at the same length scale as the experiment, and again a) increasing all dimensions by a factor of* $\sqrt{2}$ *or b) decreasing all dimensions by a factor of* $\sqrt{2}$*. Black traces show the simulation for the experimental dimensions, where are the colored traces are the rescaled simulations.*

References

(1) Pastor, I.; Vilaseca, E.; Madurga, S.; Garcés, J. L.; Cascante, M.; Mas, F. Diffusion of α-Chymotrypsin in Solution-Crowded Media. A Fluorescence Recovery after Photobleaching Study. *J. Phys. Chem. B* **2010**, *114* (11), 4028–4034. https://doi.org/10.1021/jp910811j.
